# Uniaxial Compressive Behavior of Concrete Columns Confined with Superelastic Shape Memory Alloy Wires

**DOI:** 10.3390/ma13051227

**Published:** 2020-03-09

**Authors:** Chenkai Hong, Hui Qian, Gangbing Song

**Affiliations:** 1 Faculty of Mechanical Engineering and Mechanics, Ningbo University, Ningbo 315211, China; 2 Zhejiang Engineering Technology Research Center of Civil Engineering Industrialized Construction, Ningbo University of Technology, Ningbo 315211, China; 3 School of Civil Engineering, Zhengzhou University, Zhengzhou 450001, China; 4 Department of Mechanical Engineering, University of Houston, Houston, TX 77204, USA

**Keywords:** shape memory alloy, superelasticity, concrete columns, uniaxial compression behavior

## Abstract

Superelastic shape memory alloy (SMA) exhibits the ability to undergo large deformations before reverting back to its undeformed shape following the removal of the load. This unique property underlies its great potential in the seismic design and retrofitting of structure members. In this paper, superelastic SMA wires were utilized to confine concrete cylinders to enhance their axial compressive behavior. The axial carrying and deformation capacities of SMA-confined concrete cylinders are assessed by uniaxial compression testing on a total of eight SMA-confined concrete columns and one unconfined column. The influence of the amount of SMA and the prestrain level of SMA wires, as well as the reinforcing mode, on the axial carrying and deformation capacity of confined concrete columns were considered. The analysis focuses on the axial carrying capacity and deformation performance of concrete columns reinforced with superelastic SMA under different loading conditions. Based on the experimental data and analysis results, it is found that superelastic SMA wires can increase the axial loading capacity and enhance deformation performance of concrete columns. Under the same loading condition, the ultimate bearing capacity of SMA-confined concrete columns increases as the increasing of the amount of SMA wire. The results of this study verify the effectiveness of superelastic SMA in enhancing the loading capacity and deformation behavior of concrete cylinders.

## 1. Introduction

Civil infrastructures are often operated in environments with adverse factors, such as corrosion [1,2], vibration and fatigue [3,4], impacts [5,6,7,8], seismic excitations [9,10,11], strong wind or hurricane [12], among others, and these adverse factors often result in component and/or structural damages, which should be detected and repaired in time to prevent catastrophic events from happening [13]. Structural health monitoring (SHM) [13,14] and damage detection [14,15,16,17,18] and structural retrofit and repair [19,20] have been developed to address these two issues. Concrete columns are commonly used in civil infrastructures, and SMH and retrofit of concrete columns have been actively researched. In particular, retrofitting and repair concrete columns receives increasing attention.

Experimental tests conducted by Andrawes and Shin [20], as well as the damage investigation and analysis of engineering structures in the Wenchuan earthquake and the East Japan earthquake, show that concrete columns offer good compression and stiffness performance, however poor deformability. A sharp increase of the external load applied to the concrete columns during an earthquake motion, can easily result in brittle fractures due to that the columns reached the ultimate carrying capacity. The safety and reliability of the entire structure may then be threatened, and more serious damage such as total collapse could occur. Therefore, it is of great significance to find effective methods to enhance the bearing and deformation capacities of the concrete columns.

Previous studies have shown that confinement constraints can effectively improve the axial compression performance of concrete columns [21,22,23]. Due to the good ductility of the materials, such as steel, fiber-reinforced polymer (FRP), shape memory alloy (SMA), the concrete column can obtain corresponding deformability and attain the ability to withstand large loads after reaching the ultimate bearing capacity [24,25,26,27,28]. Thus, the concrete structure has a stronger deformability and avoids overall failure or collapse damage.

Shape memory alloy (SMA) is a new smart material with functions both of sensoring and actuating [29]. The unique shape memory effect and superelastic properties (with a recoverable deformation of greater than 8%) provide SMA with the ability to enhance the compression performance of concrete columns [30]. The main advantage of SMA is that prestress can be applied through temperature phase change and shape memory effect, and a large hysteretic energy dissipation and self-reset ability due to the superelastic characteristics [31]. Hence, semi-active control and active control of the structure can be achieved. In addition, compared with steel, SMA exhibits higher strength/weight ratio (or strength/volume ratio), higher corrosion resistance, and better fatigue performance under cyclic loading [32,33,34,35,36].

Recently, it has attracted increasing attentions from researchers to use the SMA material to improve the compression performance of concrete column. Andrawes and Shin [20,37] proposed a thermally triggered prestress of prestrained SMA spirals as a means of applying active confinement to concrete columns. And the experimental and numerical study showed that under horizontal cyclic loading, the column reinforced with the SMA spirals was able to sustain larger force and drift and dissipate more hysteretic energy compared to that with glass fiber-reinforced polymer (GFRP) only. Choi et al. [23,24,38] used heated pre-elongated martensite SMA wire and tightly wound austenitic SMA wires to obtain prestressed reinforced concrete (RC) cylinders, and studied their axial compression behavior. The test results show that, although the martensitic wires increased the ultimate strength by about only 7% and the ultimate strength of concrete cylinders remained the same as that of plain concrete cylinders due to the post-tensioning stress on austenitic SMA wires was not introduced, but the failure strain of SMA wires RC cylinders increased significantly, by up to 20%, and the energy dissipation capacity improved. Furthermore, by applying the same rate in a volume configuration, the enhanced bearing capacity of the SMA concrete columns was similar to steel casing reinforcement.

Chen [39] conducted an experimental study on this new confinement technique using SMA spirals as internal transverse reinforcement in square columns. The experimental results demonstrate that, compared with traditional steel transverse reinforcement, SMA spirals effectively delay the longitudinal rebar buckling and significantly reduce strength degradation. Chen [40] demonstrated that SMA-confined concrete has greater strength and ductility than steel-based passive RC. Both Shin [41,42] and Chen [25,43] reported that SMA confinement with prestress heat treatment effectively improves the ductility and seismic behavior of vulnerable RC columns over fiber-reinforced polymer (FRP). Park et al. [44] and Pratik et al. [45] applied axial cyclic loading to concrete columns reinforced by prestressed martensitic SMA and steel casing, respectively. The results show that the skeleton curve of the concrete columns reinforced by martensitic SMA is similar to the plastic strain curve of concrete columns reinforced by steel casing In addition, in the process of repeated loading, the hysteretic feature of SMAs allows the concrete columns to undergo larger circumferential strain and consume more energy than steel casing RC columns. Furthermore, Aliakbar et al. [46] also applied a confining pressure to two types of strength (normal-strength concrete (NSC) and high-strength concrete (HSC) of concrete columns based on the effect of the temperature on the recovery stress of SMA. The compression test results of SMA-confined concrete specimens show that an increase in the prestrain level leads to significantly increases the compressive strength and corresponding axial strain. The 9.5% pre-strained SMA spirals confined normal-strength concrete and high-strength concrete specimens exhibit 2.1 and 3.4 times of the strength enhancement, respectively, and reveal 1.9 and 2.3 times of the strain enhancement, respectively, compared to those of the unstrained specimens.

The superior performance of SMA is also applied to the enhancement of other structures and approaches. Saiidi et al. [47] experimentally tested the ability of superelastic SMA reinforcement to recover deformations in concrete beams under cyclic loading. The results showed that the average residual displacement in the superelastic SMA reinforced beams was less than 1/5 of that of the steel reinforced beams. Abdulridha et al.’s research [48] also proved that SMA bars can well limit the residual displacement and crack width of the beam. Rojob et al. [49] and Michels et al. [50] anchored iron-based SMA bars inside concrete beams and applied a prestressing force through heat treatment. The results revealed a significant increase in the load capacities and ductile tensile behavior. Alam and Youssef [51,52] applied superelastic SMA rebars to reinforce beam–column joints under reversed cyclic loading and found that the residual displacements was even negligible in the SMA–RC beam–column joint compared to that of the conventional steel–RC beam–column joint. In addition, SMA–RC beam–column joint’s plastic hinge region away from the column face to a distance of approximately half of the beam-depth more than that of steel–RC beam–column joint, which resulted in higher energy dissipation. Shajil et al. [53] compared the load-deformation characteristics of concrete beams reinforced with the superelastic SMA fibers and the steel fibers under cyclic loading and reported that the superelastic SMA fibers reinforced concrete exhibited significant self-centering characteristics in comparison to the steel fibers in the post-cracked conditions. Kim et al. [54] applied short SMA fiber-reinforced cementitious composites (SMA-FRCC) to concrete columns. Heat treatment noticeably increased the Young’s modulus of the SMA-FRCCs under tension due to the shape-memory effects. Nehdi and Ali [55] conducted numerical and experimental investigations and found that incorporating SMA fibers into engineered cementitious composites yielded superior impact resistance. Wang et al. [56] introduced the use of SMA bolts to steel columns. They found that the steel columns equipped with SMA bolts showed a satisfactory self-centering and energy dissipation capabilities under bidirectional loading and multi-earthquake loading. It is clear from the existing research results that SMA is an excellent material for reinforcing concrete members. However, the shape memory effect of thermally activated martensitic SMA wire have been utilized in most of studies to constrain concrete columns owing to the difficulty to apply prestress with different levels on superelastic SMA, which leads to a large prestress loss of the SMA wire, the difficulty of final prestrain measurement, and the limited lateral restraint effect. The research of utilizing austenite SMA to directly impose active constraints on concrete columns is rare. Therefore, experimental studies are needed to explore the behavior and investigate the feasibility of austenitic superelastic SMA actively constrained concrete columns.

This paper presents a new practical method using superelastic SMA wires with adjustable pre-stressing force levels, to achieve confinement function for concrete columns. In addition, the experiments of SMA wires and concrete columns confined with prestrained superelastic SMA wires are discussed in detail to analyze the mechanical properties of SMA wires and axial load carrying capacity mechanism and mechanical properties of columns. It is hoped that the results of this research can promote the development of construction technique of superelastic SMA active restraint concrete member and contribute to the improvement of existing concrete member restraint technique.

## 2. Materials

### 2.1. Superelastic SMA Wires

In this study, homoeothermic superelastic NiTi SMA wires with a diameter of 1.2 mm were used (TN3-type, produced by Xi’an Saite Metal Material Development Co., Ltd., Xi’an, China). The chemical composition of the wires was Ni-49.1Ti (at.%), and the reverse martensite transformation temperature (Af) was −10 °C.

**Figure 1:**
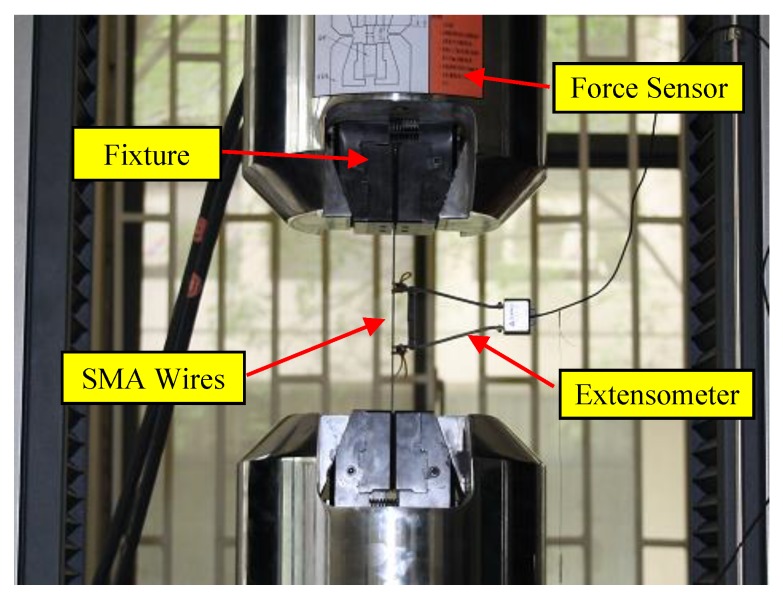
Tensile test of shape memory alloy (SMA) material.

**Figure 2:**
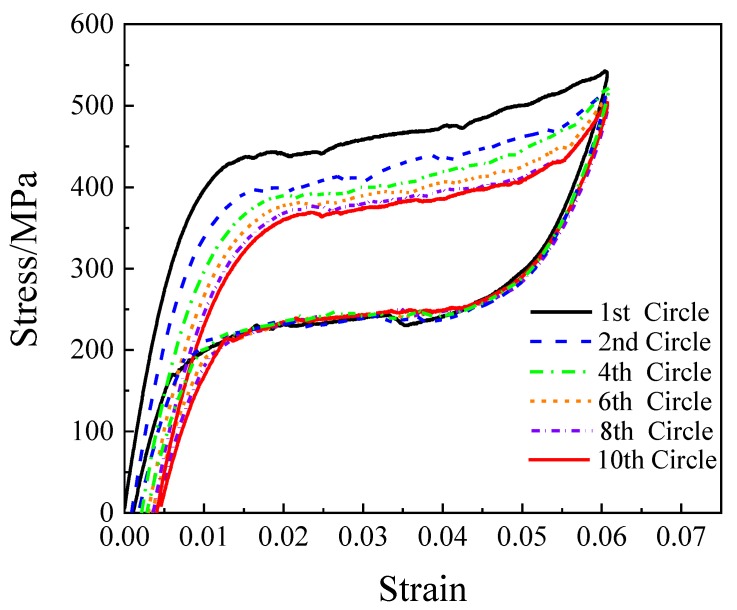
Stress–strain curves of SMA wire.

To determine the mechanical properties of the SMA wires, cyclic loading tests were conducted using the CMT universal material testing machine (Shenzhen Suns Technology Stock Co., Ltd., Shenzhen, China). The test setup is shown in Figure 1. A loading rate of 0.0005/s and loading amplitude of 6% were applied. Figure 2 shows the stress-strain relationship curves of SMA wire under 10 cyclic load. The results show that the superelastic SMA wires have a stable mechanical yield point and sustained force performance after yielding. As the number of loading cycles increases, the hysteresis curve becomes stable. Table 1 shows the mechanical properties of 1.20 mm SMA wire materials used in the test. The yield strength of the 1.20 mm SMA wires is 442.32 MPa, the strength under 6% strain is 542.10 MPa, and the elastic modulus is 247.94 GPa.

### 2.2. Mechanical Properties of Fiber Reinforced Polymer (FRP)

Carbon Fiber-reinforced Polymer (CFRP) is widely utilized in concrete structure reinforcement owing to its advantages such as light weight, high strength, corrosion resistance, and ease of construction [57,58]. When applied to reinforcing compressive members, CFRP is usually wound around concrete columns laterally, and the bearing capacity of concrete columns increase by lateral restraint of CFRP. Table 2 provides the mechanical characteristics of the FAW200-type CFRP used in this test [59].

### 2.3. Mechanical Properties of Concrete

Concrete material with a strength grade of C30 (the Chinese Code for Design of Concrete Structures (GB 50010-2010) [60]) was used in this study. A set of three 150 × 150 × 150 mm cubes was produced for strength testing. After 28 days’ cure under the same standard curing conditions as the columns, the average cubic compressive strength was 36.8 MPa, measured by the universal material testing machine.

## 3. Experiments Test Setup and Procedure

**Figure 3:**
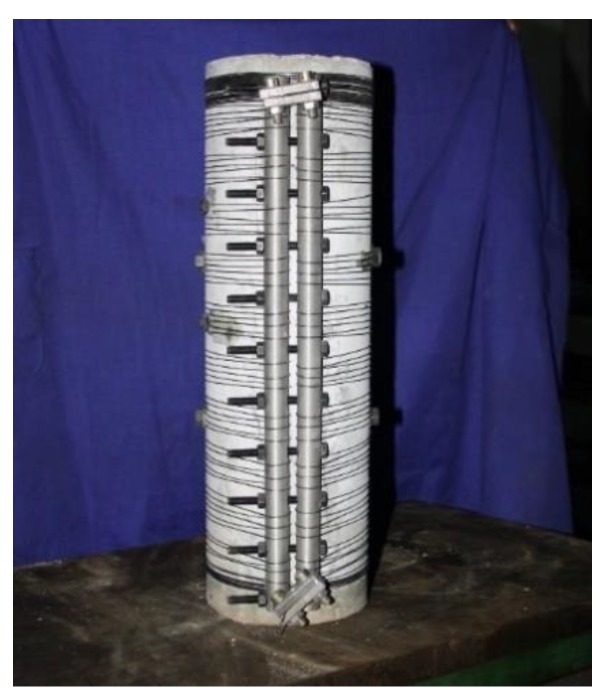
A SMA reinforced concrete cylinder with a wire tensioning and anchoring device.

Figure 3 shows a SMA reinforced concrete column specimen. The column specimens are 150 mm in diameter and 500 mm in height. The specimen was winded with prestressed Superelastic NiTi SMA wires with a diameter of 1.2 mm. In the test, a tensioning and anchoring device was proposed to apply constraints on the concrete column. This device comprises two steel cylindrical anchorages, high-strength bolts, and two fixed SMA wire grips. The SMA wire was closely wound around the cylinders and fixed to the jig and a torque wrench was used to tighten the high-strength bolts synchronously and ensure that the SMA wire prestrain was applied uniformly. The prestrain was controlled by the amount of indentation between the prestressed anchors, which was measured by a Vernier caliper.

To examine the effect of SMA amount, SMA pre-strain levels, and reinforcement modes on the axial mechanical properties of the specimens, the failure characteristics and performance of concrete columns under axial compression in the limit state were observed and the impact on the ultimate bearing capacity and the vertical/lateral deformation were identified. Three different SMA wires reinforcement spacings were applied to the cylindrical cylinders (8.0, 4.0, and 2.5 mm) and four different prestrain levels of SMA wires (0%, 1%, 2%, and 4%) were adopted. Specimens with superelastic SMA wires and FRP composite cloth were examined as the two reinforcement modes. Another specimen was designed to determine the ultimate load of an unreinforced cylinder. The test specimens are summarized in Table 3.

**Figure 4:**
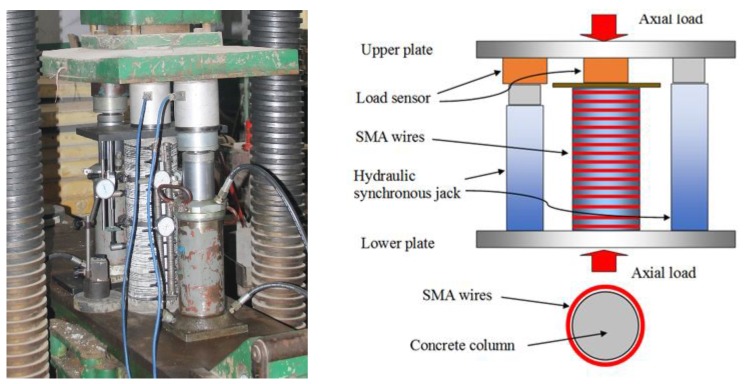
Axial compression test device and structure diagram.

The cylinders were tested on 5000 kN axial compression testing machine (manufactured by Changchun Material Testing Machine Factory, Changchun, Jilin, China), as shown in Figure 4. The experiments were carried out in the force control mode. Failure criterion for the concrete cylinder specimens occur due to the crush of concrete cylinder or a sudden decline of carrying capacity caused by the fracture of superelastic SMA wires.

According to the relevant provisions of the Standards for Testing Methods of Concrete Structures (GB 50152-2012) [61], the load control method was used to grade the loading. Under these standards, when the test load is less than 60% of the estimated ultimate load of the concrete cylinder specimen, the graded load is taken as 10% of the estimated ultimate load; when the specimen load is greater than 60% of the estimated ultimate load of the concrete cylinder, the graded load is taken as 5% of the estimated ultimate load until the specimen reaches the ultimate load. To fully deform the test piece, it should be loaded with a voltage for not less than 10 min after each stage of loading has been completed. After the bearing capacity begins to decrease, the inlet and the squeegee of the test machine and the synchronous jack should be controlled so that the strain of the test piece changes slowly and steadily until it is destroyed.

The failure criterion of concrete cylinder specimens is that the bearing capacity of the specimen suddenly drops due to the cylinder concrete being crushed or the superelastic SMA wires used as reinforcement break [61].

**Figure 5:**
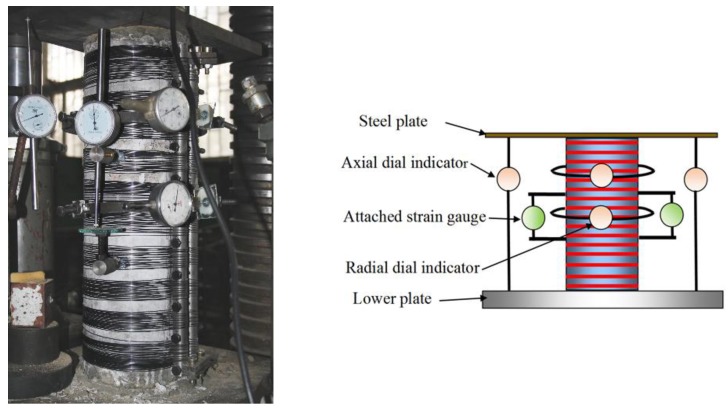
Dial indicator arrangement and test schematic.

Throughout the test process, the bearing capacity of the concrete cylinder was mainly determined by force sensors (Shanghai Zhaohui Pressure Instrument Co., Ltd., Shanghai, China). The dial gauge measured the overall and partial vertical deformation, as well as the lateral deformation of the concrete cylinder. Since the strain gauge cannot be attached to the reinforced cylinder, two vertical dial indicators with appurtenances are configured in the middle of the concrete cylinder to measure the local deformation in the middle of the cylinder within 150 mm and then strain can be calculated, as shown in Figure 5. In addition, the range and repeatability of dial indicators is 50 mm and 0.001 mm, respectively.

## 4. Experimental Phenomena and Test Results Analysis

### 4.1. Experimental Phenomena

**Figure 6:**
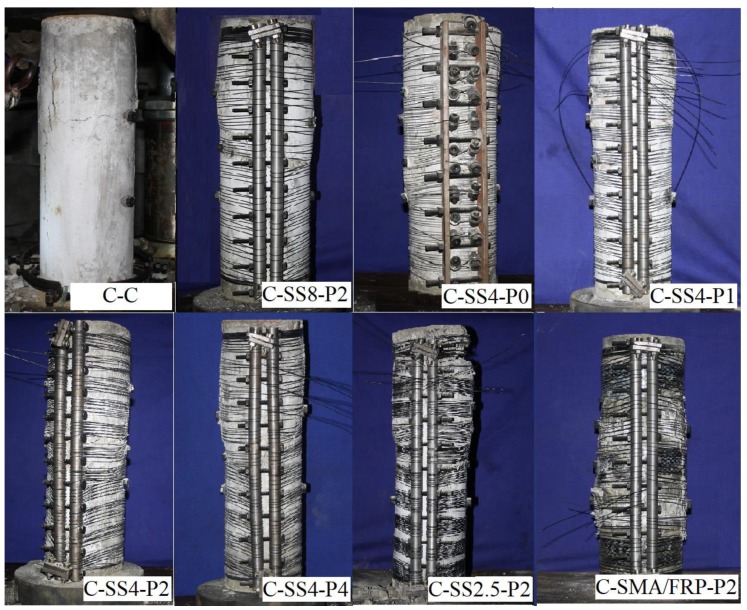
Superelastic SMA reinforced concrete cylinders failure patterns.

The typical failure modes of concrete cylinders under compression were illustrated in Figure 6. The plain concrete cylinder experienced rapidly failure after reaching its ultimate bearing capacity. The main crack expanded and extended most significantly, reaching both ends of the specimen and intersecting with the transverse cracks. The superelastic SMA RC cylinders showed significant inelasticity after the first cracks appeared, and the carrying capacity continued to increase. After reaching the ultimate carrying capacity, some crack development occurred in the specimens, and deformed growth and central bulging was observed, albeit with steady loading. The SMA confined specimen failure was due to concrete crushing, with the SMA wires rupturing at the bulging points with a popping sound and the carrying capacity decreasing rapidly. Ultimately, inclined main cracks ran through the middle of the cylinder, and the deformation was concentrated here. The ultimate carrying capacity of the SMA-FRP RC cylinder was reached before the FRP fractured, and then the bearing capacity briefly plateaued. Vertical cracks were observed throughout the entire cylinder in the middle of the specimen, and obvious bulging was apparent where the SMA wire fractured, resulting in a loss of carrying capacity of the specimen.

### 4.2. Test Results Analysis

**Figure 7:**
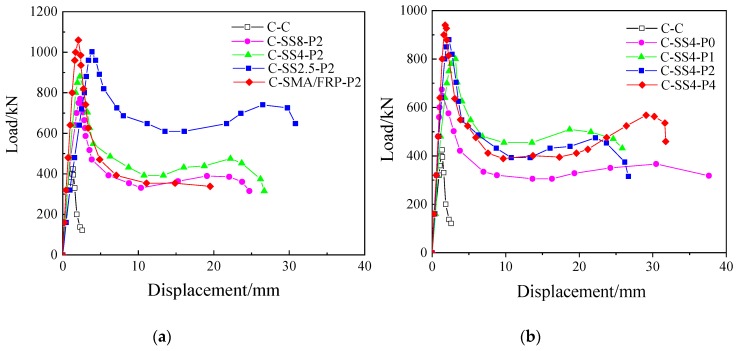
Axial load-displacement curves of concrete columns: (**a**) Axial load-displacement curve of concrete cylinders with different SMA wires reinforcement amount and (**b**) Axial load-displacement curve of concrete cylinders with different prestrain level of SMA wires.

Figure 7 shows the axial load-displacement curves of concrete columns. Figure 7a prefers to the axial load-displacement curve of concrete cylinders under different amount of SMA wires. Figure 7b indicates the axial load-displacement curve of concrete cylinders with different prestrain level of SMA wires. As can be seen in Figure 7, the SMA reinforced cylinders experienced five stages: (1) the elastic stage, (2) the ultimate bearing stage, (3) the rapid descent phase, (4) the stability and strengthening growth stage, and (5) the reverse strengthening effect stage. Among them, stage 5 is the most prominent. The SMA+FRP reinforced cylinders underwent the elastic stage, ultimate bearing stage, rapid decline phase, and stabilization phase.

Based on the test data, the carrying capacity and strain values of superelastic SMA RC cylinders are presented in Table 4. Data analysis indicates that, with the same prestrain level *α* = 2%, the ultimate bearing capacity of C-SS8P2, C-CS4P2, and C-SS2.5P2 compared with the plain concrete cylinder C-C was increased by up to about 80%, 105%, and 135%, respectively. The bearing capacity of the concrete cylinders exhibited approximately linear growth with respect to the reinforcement amount of superelastic SMA. With *λ* = 0.180, the ultimate bearing capacity of C-SS4P0, C-SS4P1, C-SS4P2, and C-SS4P4 were increased by about 60%, 90%, 105%, and 120%, respectively, compared with the C-C concrete cylinder. This indicates that the reinforcing effect of the prestrained superelastic SMA wires enhances the ultimate bearing capacity of concrete cylinders to a remarkable degree. However, the enhancement weakened as the prestrain level increased.

For the same reinforcement configuration and prestrain, C-SS2.5P2 and C-SMA/FRP-P2 were reinforced with superelastic SMA and superelastic SMA+FRP, respectively. The ultimate bearing capacity, compared with C-C, increased by 139.8% (SMA-S2.5P2) and 149.4% (C-SMA/FRPP2). Both forms of reinforcement significantly improve the ultimate bearing capacity of concrete cylinders and compared with superelastic SMA wire, the reinforcement effect of superelastic SMA+FRP composite is slightly better.

### 4.3. Axial and Lateral Strain Relationship of Concrete Column

Based on the phenomenon observation of loading process and the analysis of the specimens failure mode, it is found that deformation and cracking occurred mainly in the middle of the specimens. In this section, the relationship and comparison among axial deformation in the middle of the specimens (measured by two vertical dial indicators on both sides and average value is adopted), radial deformation (measured by the radial dial indicators at 0.5 h), and the bearing capacity were analyzed.

**Figure 8:**
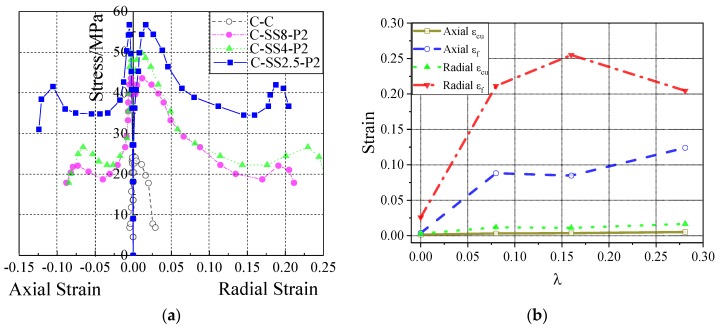
Axial stress–strain relation comparison of concrete cylinders with different reinforcement amount of SMA wires: (**a**) Axial stress–strain curves of concrete cylinders with different reinforcement amount of SMA wires; (**b**) Comparison of axial and lateral peak strain and failure strain of concrete cylinders with different reinforcement amount of SMA wires.

Figure 8 shows the relationship curves between the axial stress and axial/radial strain of concrete cylinders reinforced with 2% prestressed SMA wires. From the Figure 8a, we can see that compared to common column (C-C), the bearing capacities of the SMA confined columns were greatly enhanced. Not only did the ultimate stress increase with the amount of SMA wire, but also the axial strain and lateral strain also showed increasing trends with the increasing amount of SMA wires. As the most reinforced specimen, the axial *ℰ*_cu_, *ℰ*_f_, and lateral *ℰ*_cu_, *ℰ*_f_, of SMA-S2.5P2 increased by factors 3.6, 34.4, 5.9, and 7.9 compared with specimen C-C.

**Figure 9:**
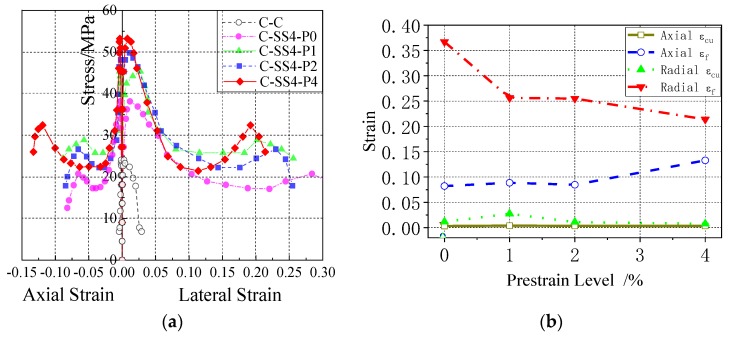
Axial and lateral stress–strain relation comparison of SMA wire reinforced concrete cylinders with different prestrain levels: (**a**) Axial and radial stress-strain curves of SMA reinforced concrete cylinders with different prestrain levels; (**b**) Comparison of axial and lateral peak strain and failure strain of concrete cylinders with different prestrain levels.

Figure 9 shows the relationship curves between the axial stress and axial/radial strain of concrete cylinders reinforced SMA wires with different prestrain levels. From the figures, we can see that, for the same amount of reinforcement, an increase in the prestrain level of the SMA wires produces a corresponding increase in the ultimate axial stress of the concrete cylinder. Additionally, the peak axial strain and failure strain showed a significant increasing trend, while the peak lateral strain and failure strain declined. This was because the increased prestrain enhanced the side constraints of the concrete cylinders, which widened the deformation area and made the distribution more uniform. In contrast, an increase in the amount of prestrained superelastic SMA wire led to the strengthening phase occurring earlier and the subsequent deformation capacity decreasing slightly.

**Figure 10:**
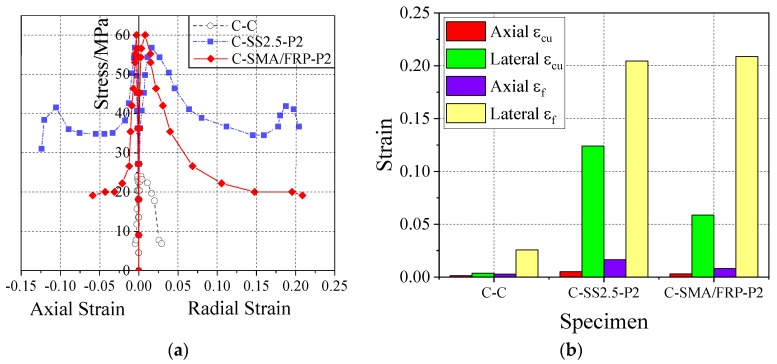
Axial and lateral stress–strain relation comparison of concrete cylinders with different reinforcement mode, which involves SMA and SMA/FRP: (**a**) Axial and radial stress-strain curves of concrete cylinders with different reinforcement mode, which involves SMA and SMA/FRP and (**b**) Comparison of axial and lateral peak strain and failure strain of concrete cylinders with different reinforcement mode, which involves SMA and SMA/FRP.

Figure 10 shows that the two different reinforcement forms of SMA and SMA/FRP increase the axial stress, axial strain, and lateral strain, and improve the axial bearing of the concrete cylinders. Contrasting C-SS2.5-P2 and C-SMA/FRP-P2, it is found that although the ultimate stress of the SMA-enhanced cylinder is slightly smaller than that of the SMA/FRP reinforced cylinder, its follow-up stress and axial/lateral strain development are better. In addition, the deformation of the SMA-reinforced cylinder exhibits a more uniform distribution than the SMA/FRP reinforced cylinder. Thus, the deformation capacity and ductility of the SMA RC cylinder are much better than in the SMA/FRP specimen.

Figure 8a, Figure 9a, and Figure 10a show that, in the same test specimen, SMA wires increases the lateral strain of the concrete cylinder more than the axial strain. This is especially true in the elastic stage, ultimate bearing stage, and rapid descent phase, consistent with the test results obtained by Chen et al. [25]. Combined with the analysis of the experimental phenomena, applying of SMA prestressing is very helpful for the full development of fine oblique cracks in concrete cylinders after exceeding the elastic strain limit. Therefore, prestressed SMA wires can greatly improve the lateral deformation capacity of concrete cylinder.

Because of the excellent constraining ability and superelastic deformation ability of SMA wires, the axial *ℰ*_cu_ of all SMA-confined test specimen (C-SS8-P2, C-SS2.5-P2) is more than twice that of C-C, and the axial *ℰ*_f,_ is 22.8–36.9 times as high. The lateral *ℰ*_cu_ is almost 4 times that of C-C, except for sample C-SS4-P4, which is only 2.7 higher than the unreinforced specimen because of the high prestrain. The lateral *ℰ*_f_ is almost eight times higher than that of C-C. Prestressed SMA wires considerably enhance the axial and lateral deformation capacity of concrete cylinders, significantly improving their survivability under the action of disasters.

## 5. Design and Calculation of Ultimate Axial Bearing Capacity of Superelastic SMA RC Columns

To facilitate the calculation of the axial compressive bearing capacity of concrete cylinders confined by prestrained superelastic SMA wires, the following simplified assumptions are proposed on the premise of ensuring the calculation accuracy:

(1) During the prestraining process, the superelastic SMA wire changes uniformly under tension, and the constraint effect on the cylinder surface is also uniform. The differences in the prestrain level on the cylinder surface due to the friction between the concrete and the SMA wires can be neglected;
(2) The axial compressive strength of concrete cylinders constrained by prestrained superelastic SMA wires is composed of the axial compressive strength of the unconstrained concrete cylinders and the increased axial compressive strength of the concrete cylinders enhanced by the lateral restraint effect of the superelastic SMA wires. The axial compressive strength of SMA constrained concrete cylinders can be obtained by linearly superposing the axial compressive strength of unconstrained concrete cylinder and the increased axial compressive strength of the concrete cylinders enhanced by the lateral restraint effect of the superelastic SMA wires;
(3) The axial load on the cylinder is fully borne by the concrete, and the superelastic SMA wire does not bear the axial load;
(4) The SMA wires are always in a tensioned state, and in reliable contact with the concrete columns. The two items work together and deform together, which is also proved by experimental phenomena. Hence there is no relative slippage during the loading process.

### 5.1. Calculation of Compressive Strength of Concrete Columns Confieded by Prestressed Superelastic SMA Wires

The compressive strength of concrete columns increases because of the strong active confinement restraining force generated by prestressed superelastic SMA wires. The lateral expansion and crack development are suppressed, and the core concrete is in a three-way compression state. Therefore, the constraining force of the SMA wires is an important factor. The compressive strength of the confined concrete is mainly composed of the unconstrained concrete strength and the improved constrained action strength. Referring to Mander Constrained Concrete Constitutive Model [62,63], the axial compressive strength of concrete columns constrained by prestrained superelastic SMA wires can be written as:

(1) $$f_{cc}=f_{c}+k_{l}f_{l}$$

where, *f_cc_*: Axial compressive strength (MPa) of concrete cylinder confined by superelastic SMA wire; *f_c_*: Axial compressive strength (MPa) of the unconstrained concrete cylinder; *k_l_*: Lateral constraint stress effect coefficient; *f_l_*: Average lateral compressive stress of SMA wire (MPa).

**Figure 11:**
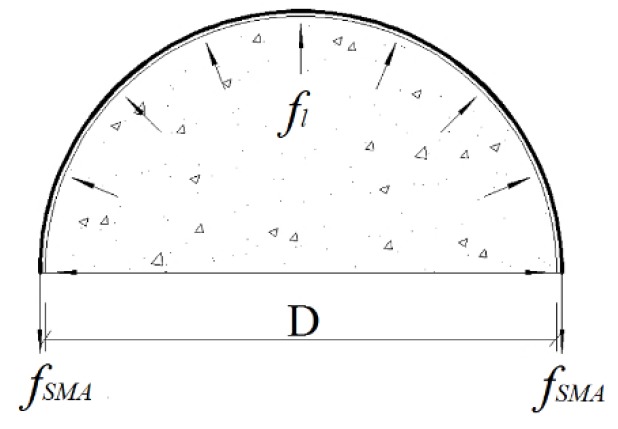
Superelastic SMA wire reinforced concrete cylinder.

Figure 11 shows the confining effect of the superelastic SMA wires on the concrete column. Assuming that the SMA wires are uniformly distributed in the reinforced range of the concrete column, the distribution of the lateral restraint force $f_{l}$ is also uniform on the column surface. The deviation is corrected by the lateral stress coefficient $k_{l}$. Thus, from the equilibrium condition of the limit stress state of the section, we obtain:

(2) $$\int_{0}^{\pi }f_{l}s\frac{D}{2}d\theta \cdot sin\theta =2f_{ySMA}A_{SMA}$$

where, *f_SMA_*: Phase transition stress (MPa) of the superelastic SMA wire (equivalent to the yield stress of the steel); *A_SMA_*: Cross-sectional area of the superelastic SMA wire (mm^2^),$\;A_{SMA}=\pi d_{SMA}^{2}/4$; *s*: Reinforcement spacing (mm); *θ:* Angle; *D*: Diameter of the concrete cylinder (mm).

Integrating Equation (2) gives:

(3) $$f_{l}=\frac{2f_{ySMA}A_{SMA}}{Ds}$$

The axial compressive strength of concrete columns constrained by prestrained superelastic SMA wires can be expressed as:

(4) $$f_{cc}=f_{c}+k_{l}\frac{2f_{ySMA}A_{SMA}}{Ds}$$

The contribution of the lateral restraint stress of the SMA wires to the compressive strength of the concrete cylindrical axis can be expressed as:

(5) $$k_{l}f_{l}=f_{cc}-f_{c}$$

(6) $$f_{cc}-f_{c}=k_{l}f_{l}=\frac{k_{l}}{s}\cdot \frac{2f_{SMA}A_{SMA}}{D}$$

where $\frac{2f_{SMA}A_{SMA}}{D}$ is a fixed value, and let $k_{l}^{\prime }=\frac{k_{l}}{s}$, the unit is mm^−1^.

(7) $$f_{cc}-f_{c}=k_{l}^{\prime }\cdot \frac{2f_{SMA}A_{SMA}}{D}$$

**Figure 12:**
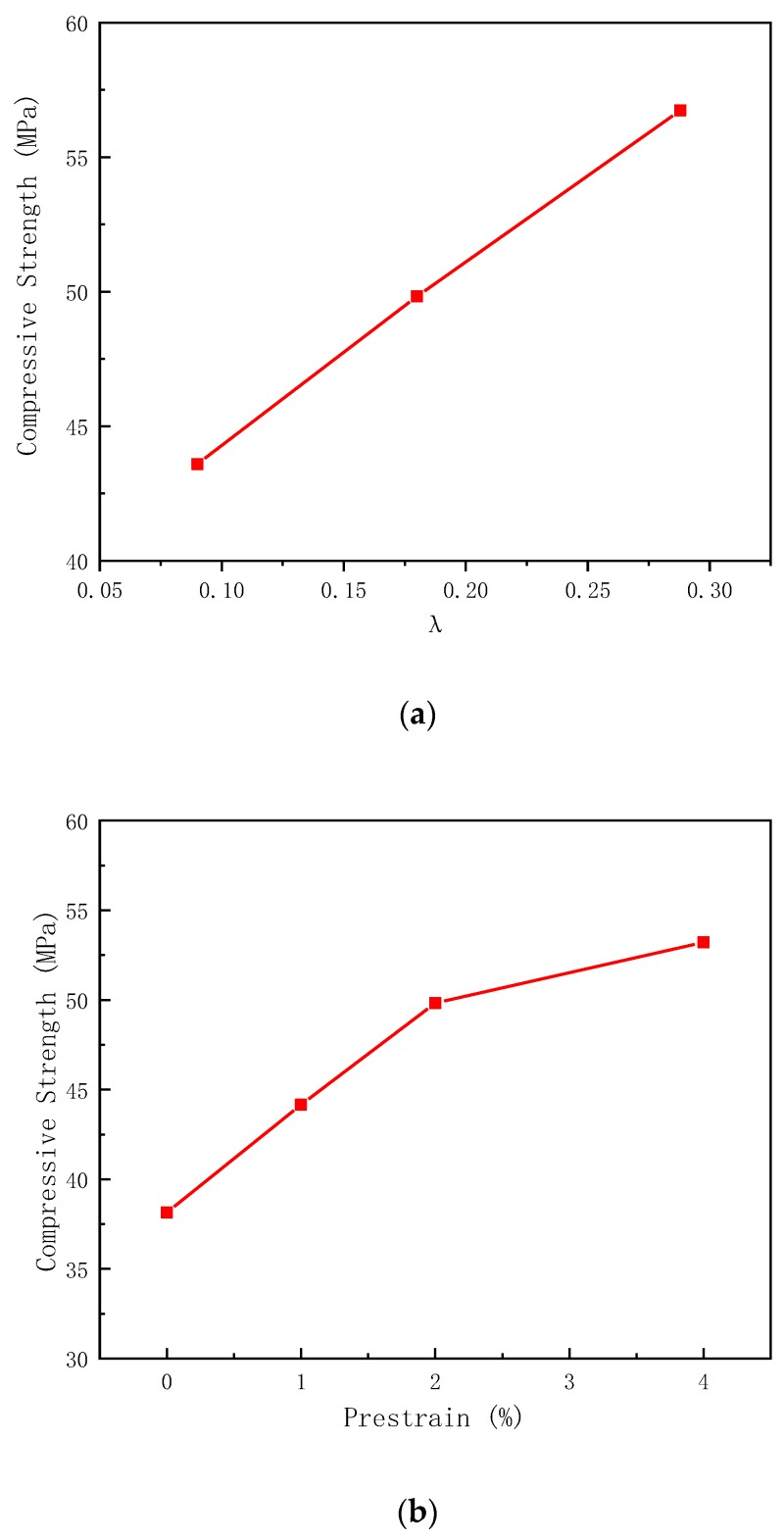
Ultimate compressive strength of SMA reinforced columns with different reinforcement amount and different prestraining levels: (**a**) Ultimate compressive strength of test specimens C-SS8-P2, C-SS4P-2, and C-SS2.5-P2 with different reinforcement amount; (**b**) Ultimate compressive strengthC-SS4-P0, C-SS4-P1, C-SS4-P2, and C-SS2.5-P4 with different prestraining levels.

Figure 12 shows the ultimate compressive strength of SMA reinforced columns with different reinforcement amount and different prestressing levels. As can be seen in Figure 12, data analysis shows that $k_{l}^{\prime }$ in the SMA wires increases approximately linearly with the increase of *λ*; as the prestrain level increases, the growth of $k_{l}^{\prime }$ slows down. According to the variation of the contribution of lateral constrained stress to the axial compressive strength of the concrete columns, it is assumed that the functional relationship between $k_{l}^{\prime }$, *λ*, and *α* is as follows:

(8) $$k_{l}^{\prime }=\left(a_{1}\lambda +a_{2}\right)\left(b_{1}\alpha^{2}+b_{2}\alpha +b_{3}\right)$$

where $a_{1}$, $a_{2}$, $b_{1}$, $b_{2}$, and $b_{3}$ are undetermined constants.

Consider the influence of *λ* and *α* on $k_{l}^{\prime }$ in Equation (6). According to the data in Table 5, the five undetermined constants and $k_{l}$ can be determined using nonlinear regression.

(9) $$k_{l}^{\prime }=\left(1.76\lambda +0.36\right)\left(-21.7\alpha^{2}+1.72\alpha +0.03\right)\times 10^{6}$$

where 0.090 ≤ *λ* ≤ 0.288 and 0 ≤ *α* ≤ 0.04.

The component (*f_cc_* − *f_c_*)_1_ can be obtained by substituting Equation (9) into Equation (7), that is, the contribution of the SMA wire lateral restraint stress to the compressive strength of the concrete column axis $k_{l}f_{l}$. A comparison with the test values is presented in Table 6, and the maximum difference between the two does not exceed 3.4%.

The axial compressive strength of the concrete columns restrained by prestressed superelastic SMA wires is:

(10) $$f_{cc}=f_{c}+\left(1.76\lambda +0.36\right)\left(-21.7\alpha^{2}+1.72\alpha +0.03\right)\times 10^{6}\cdot \frac{2f_{ySMA}A_{SMA}}{D}$$

where 0.090 ≤ *λ* ≤ 0.288 and 0 ≤ *α* ≤ 0.04.

Based on the calculation model for the compressive strength of concrete columns confined by prestrained superelastic SMA wires, ultimate axial bearing capacity of concrete cylinders restrained by prestrained superelastic SMA wires can be:

(11) $$N_{u}=f_{cc}\cdot A=\left(f_{c}+\left(4.0968\lambda +0.8321\right)\left(-931.8192\alpha^{2}+73.9154\alpha +1.3261\right)\frac{2f_{ySMA}A_{SMA}}{D\cdot s}\right)\cdot A$$

where 0.090 ≤ *λ* ≤ 0.288 and 0 ≤ *α* ≤ 0.04, the column cross-sectional area is $A=\pi D^{2}/4$.

### 5.2. Comparison of Calculated and Recorded Ultimate Axial Bearing Capacities

To verify the rationality of Equation (10), the axial bearing strength of concrete cylinders confined by prestrained superelastic SMA wires was calculated using Equation (10). The results are compared with the experimental values reported in previous research and in this paper in Table 6.

The errors of most test specimens do not exceed 5%, and only four test specimens (Ma3, Ma4-II, NiTi-2, and NiTiNb-2) have errors of 6–9%. The focus of this article is to make a useful exploration of the calculation method and recommended formula for the axial bearing capacity of SMA-constrained concrete columns, which is expected to lay the foundation for further research in the future. Therefore, although the error is large, the comparison of calculated and experimental values is still stated here as part of the research work. These results indicate that the calculated values of the specimens are in good agreement with the experimental values, and the deviation is limited. Therefore, the simplified formula in Equations (10) and (11) offers good reliability and practicality for calculating the ultimate bearing capacity of concrete columns restrained by superelastic SMA wires.

## 6. Conclusions

Research in this paper has shown that the superelastic SMA has a good reinforcing effect on the concrete columns under axial compression. It has a substantial increase in ultimate bearing capacity, while enhanc the performance of axial and radial deformation and ductility. This research provid a significant reference to improve the ability of the concrete columns to survive in the disaster. On the basis of the experimental results and discussions the following conclusions can be drawn:

(1) This paper proposes an effective method of using superelastic SMA wires to achieve active restraint and strengthen the bearing capacity of concrete columns.
(2) With the same prestrain level (*α* = 2%), an increase in reinforced amounts (*λ*) of superelastic SMA from 0.09 to 0.288 leads to an approximately linear increase of ultimate bearing capacity of SMA RC columns from 80% to 135%, compared with the plain concrete column.
(3) With the same reinforced amounts (*λ* = 0.180), an increase in prestrain level from 0% to 4% results in an remarkable increase of ultimate bearing capacity of SMA RC columns from 60% to 120%. However with prestrain level increasing, the enhancing trend weakened.
(4) Though both SMA and SMA/FRP have similar effects on strengthening the ultimate bearing capacity of concrete columns, SMA reinforced columns have better ductility.
(5) In the main deformation area of the concrete columns, the enhancing effect of SMA wires on the lateral strain is better than that in the axial strain.
(6) A practical calculation method and formula for the ultimate axial compression bearing capacity of concrete columns restrained by prestrained SMA wires is proposed.
(7) The number of test samples in this study is limited, so further research is needed on the bearing performance of superelastic SMA reinforced concrete columns. In particular, the calculation method for the bearing capacity of super-elastic SMA-constrained concrete columns will be further developed in future research.

Over all, this research provides a significant reference for improving the ability of concrete columns to survive disaster scenarios.

## Figures and Tables

**Table 1:** Mechanical properties of 1.20 mm SMA wire materials.

Diameter (mm)	Nominal Cross-sectional Area (mm^2^)	Young’s Modulus (GPa)	Yield Strength (MPa)	6% Strain Strength (MPa)
1.20	1.13	247.94	442.32	542.10

**Table 2:** Fiber-reinforced polymer (FRP) cloth material mechanical properties.

Nominal Thickness (mm)	Young’s Modulus (GPa)	Tensile Strength (MPa)	Poisson’s Ratio
0.11	290	3200	0.1062

**Table 3:** Test piece design.

Specimen	Reinforcement Spacing *s* (mm)	Configuring Feature Values *λ*	Prestrain Level *α*
C-C	-	-	-
C-SS8-P2	8.0	0.090	2%
C-SS4-P0 C-SS4-P1 C-SS4-P2 C-SS4-P4	4.0	0.180	0% 1% 2% 4%
C-SS2.5-P2	2.5	0.288	2%
C-SMA/FRP-P2	SMA@6.25+3FRP@125	0.288	2%

^1^*λ:* Characteristic value of the reinforcement configuration, *λ* = ρ·*f_ySMA_*/*f_c_*; ρ: Volumetric ratio of SMA wire, ρ = *4A_SMA_*/*(Ds)*; *A_SMA_*: Cross-sectional area of SMA wire; *D:* Diameter of concrete column section; *s:* Reinforcement spacing (mm); *f_ySMA_*: Tensile yield strength of SMA wire; *f_c_*: Concrete strength; *α:* Prestain level, *α* = *∆*/*L; ∆:* Pre-tension deformation of SMA wire; *L:* Initial length of SMA wire.

**Table 4:** Axial compressive bearing capacity and deformation of concrete cylinders.

Specimen	*N*_cu_/kN	*γ*_cu_	Axial *ℰ*_cu_	Lateral *ℰ*_cu_	*N*_f_/kN	*γ*_f_	Axial ℰ_f_	Lateral *ℰ*_f_
C-C	425	1.000	0.0014	0.0028	200	1.000	0.0036	0.0257
C-SS48P2	770	1.812	0.0031	0.0117	360	1.800	0.0881	0.2114
C-SS4-P0	674	1.586	0.0033	0.0115	318	1.590	0.0821	0.3669
C-SS4-P1	800	1.882	0.0039	0.0274	432	2.160	0.0886	0.2563
C-SS4-P2	880	2.071	0.0035	0.0109	373	1.865	0.0847	0.2549
C-SS4-P4	940	2.212	0.0034	0.0076	459	2.295	0.1328	0.2138
C-SS2.5-P2	1002	2.358	0.0051	0.0165	648	3.240	0.1240	0.2045
C-SMA/FRP-P2	1060	2.494	0.0030	0.0081	338	1.690	0.0586	0.2087

^1^*N*_cu_: Ultimate axial compressive bearing capacity of the column (i.e., maximum load in the Load–Displacement curves); *N*_f_: Failing axial compressive bearing capacity (i.e., the load when column fails which is determined by the failure criterion in the paper); *γ*_cu_: Increasing ratio of the ultimate bearing capacity; *γ*_f_: Increasing ratio of the corresponding failure load; Axial *ℰ*_cu_: Axial strain corresponding to the ultimate bearing capacity; Lateral *ℰ*_cu_: Lateral strain corresponding to the ultimate bearing capacity; Axial ℰ_af_: Axial strain corresponding to destruction load; Lateral *ℰ*_f_: Lateral strain corresponding to destruction load.

**Table 5:** Contribution of SMA wire compressive strength.

Specimen Number	*λ*	*α*	*σ*_cu_/MPa	(*f_cc_* − *f_c_*)	(*f_cc_* − *f_c_*)_1_	(*f_cc_* − *f_c_*)_1_/(*f_cc_* − *f_c_*)
C-C	-	-	24.06	-	-	-
C-SS8-P2	0.090	2%	43.59	19.53	19. 62	1.004
C-SS4-P0	0.180	0%	38.15	14.09	13.99	0.993
C-SS4-P1	0.180	1%	44.16	20.10	20.78	1.034
C-SS4-P2	0.180	2%	49.82	25.76	25.61	0.994
C-SS4-P4	0.180	4%	53.22	29.16	29.40	1.008
C-SS2.5-P2	0.288	2%	56.74	32.68	32.80	1.004

^1^*λ:* characteristic value of the reinforcement configuration, *λ* = ρ·*f_y__SMA_*/*f_c_*; ρ: Volumetric ratio of SMA wire, ρ =·*(4A_SMA_)*/*(Ds); A_SMA_*: Cross-sectional area of SMA wire; *D:* Diameter of concrete column section; *s:* Reinforcement spacing (mm); *f_ySMA_*: Tensile yield strength of SMA wire; *f_c_*: Concrete strength; *α:* prestrain level, *α* = *∆*/*L;* *σ*_cu_: compressive strength limit value of the concrete column specimen; (*f_cc_* − *f_c_*): Test value of the contribution of SMA wire lateral constraint to the compressive strength of the concrete column; (*f_cc_* − *f_c_*)_1_: calculated value of the contribution of SMA wire lateral constraint to the compressive strength of the concrete cylindrical axis column.

**Table 6:** Comparison of calculated and experimental values for concrete cylinder restrained by SMA wires.

Literature Tests	Specimen Number	*λ*	*α*	*f*_cc1_/MPa	*f_cc_*/MPa	*f*_cc1_/*f_cc_*
Literature [21]	Ma1	0.097	2.9%	28.06	27.79	1.010
	Ma2	0.097	2.9%	28.06	27.41	1.024
	Ma3	0.048	2.9%	27.72	26.02	1.065
	Ma4	0.048	2.9%	27.72	27.12	1.022
	Au1	0.097	0%	26.66	25.48	1.046
	Au2	0.048	0%	26.55	26.51	1.001
Literature [24]	Ma2-I	0.097	2.9%	28.06	27.31	1.027
	Ma2-II	0.097	2.9%	28.06	27.41	1.024
	Ma4-I	0.048	2.9%	27.72	26.61	1.042
	Ma4-II	0.048	2.9%	27.72	26.12	1.061
	Au4	0.097	0%	26.66	27.02	0.987
	Au2	0.048	0%	26.55	26.19	1.014
Literature [38]	NiTiNb-1	0.211	4.2%	45.31	45.38	0.998
	NiTiNb-2	0.211	4.2%	45.31	43.97	1.030
	NiTi-1	0.155	6.2%	38.19	38.48	0.992
	NiTi-2	0.155	6.2%	38.19	40.63	0.940
Literature [44]	NiTiNb-1	0.081	4.8%	43.10	43.50	0.991
	NiTiNb-2	0.040	4.8%	41.43	38.00	1.090
This Paper	C-SS8-P2	0.090	2.0%	43.54	43.60	0.999
	C-SS4-P0	0.180	0.0%	37.94	38.15	0.995
	C-SS4-P1	0.180	1.0%	44.71	44.16	1.012
	C-SS4-P2	0.180	2.0%	49.52	49.82	0.994
	C-SS4-P4	0.180	4.0%	53.29	53.22	1.001
	C-SS2.5-P2	0.288	2.0%	56.69	56.74	0.999

^1^*f*_cc1_: Calculated value of the bearing capacity of concrete cylinders restrained by superelastic SMA wire; *f*_cc1_/*f_cc_*: Comparison between the calculated and experimental values.
